# Advancing Mobile Neuroscience: A Novel Wearable Backpack for Multi-Sensor Research in Urban Environments

**DOI:** 10.3390/s25237163

**Published:** 2025-11-24

**Authors:** João Amaro, Rafael Ramusga, Ana Bonifácio, André Almeida, João Frazão, Bruno F. Cruz, Andrew Erskine, Filipe Carvalho, Gonçalo Lopes, Ata Chokhachian, Daniele Santucci, Paulo Morgado, Bruno Miranda

**Affiliations:** 1 Institute of Physiology, Lisbon School of Medicine, University of Lisbon, 1649-028 Lisbon, Portugal; joao-amaro@edu.ulisboa.pt (J.A.); anabonifacio@edu.ulisboa.pt (A.B.); 2 Associate Laboratory TERRA, Centre of Geographical Studies, Institute of Geography and Spatial Planning, University of Lisbon, 1649-004 Lisbon, Portugal; rafaelramusga@edu.ulisboa.pt (R.R.); paulo@edu.ulisboa.pt (P.M.); 3 NeuroGEARS Ltd., London NW1 7EA, UK; a.almeida@neurogears.org (A.A.); bruno.cruz@alleninstitute.org (B.F.C.); a.erskine@neurogears.org (A.E.); g.lopes@neurogears.org (G.L.); 4 NeuroGEARS Portugal Lda, 1600-514 Lisbon, Portugal; j.frazao@neurogears.org (J.F.); filipe@neurogears.org (F.C.); 5 Climateflux GmbH, 80337 Munich, Germany; ata@climateflux.com (A.C.); daniele@climateflux.com (D.S.)

**Keywords:** neurourbanism, wearable sensors, mobile electroencephalography (EEG), physiological monitoring, eye-tracking, urban health, real-world data collection

## Abstract

Rapid global urbanization has intensified the demand for sensing solutions that can capture the complex interactions between urban environments and their impact on human physical and mental health. Conventional laboratory-based approaches, while offering high experimental control, often lack ecological validity and fail to represent real-world exposures. To address this gap, we present the *eMOTIONAL Cities Walker*—a portable multimodal sensing platform designed as a wearable backpack unit developed for the synchronous collecting of multimodal data in either indoor or outdoor settings. The system integrates a suite of environmental sensors (covering microclimate, air pollution and acoustic monitoring) with physiological sensing technologies, including electroencephalography (EEG), mobile eye-tracking and wrist-based physiological monitoring. This configuration enables real-time acquisition of environmental and physiological signals in dynamic, naturalistic settings. Here, we describe the system’s technical architecture, sensor specifications, and field deployment across selected Lisbon locations, demonstrating its feasibility and robustness in urban environments. By bridging controlled laboratory paradigms with ecologically valid real-world sensing, this platform provides a novel tool to advance translational research at the intersection of sensor technology, human experience, and urban health.

## 1. Introduction

The 21st century has been described as the *Century of the City*, reflecting the unprecedented demographic shift toward urban living [1]. Global population growth is projected to continue for several decades, reaching approximately 10.3 billion by 2080, with the majority concentrated in urban areas [2]. While urban development has created opportunities and improved quality of life, it also generates challenges that negatively affect physical and mental health [3]. Urban residents, for example, are more likely to experience anxiety and depression compared to rural populations [4]. At the same time, exposure to restorative environments such as green or blue spaces has been shown to promote psychological recovery [5,6,7,8]. Yet the physiological and neural mechanisms underlying these associations remain poorly understood, as only a limited number of studies have examined brain and body responses that link environmental exposures to health outcomes [9].

Traditional research on human–environment interactions has relied heavily on stationary participants in controlled laboratory settings. While these methods offer strong experimental control, they often lack ecological validity. Recent advances in mobile neuroscience technologies now permit the recording of brain activity during freely moving behaviour [10], opening new possibilities for studying human experience in real-world urban contexts. The emerging fields of *Neurourbanism* (and also *Environmental Neuroscience*), embraces an interdisciplinary framework by integrating robust neuroscientific methods with environmental assessment to achieve a more objective and comprehensive understanding of how built and natural urban features influence subjective experience, behaviour as well as the physical and mental well-being [11,12].

Within this framework, wearable and mobile sensors play a central role, as they enable the direct measurement of brain and body physiological responses in naturalistic conditions. Mobile neuroimaging has emerged as a prominent set of tools for investigating cognitive and affective neural processes beyond the confines of laboratory—and particularly wihin city environments [13]. By combining such approaches with environmental sensing, researchers can obtain richer insights into the interplay between urban context and mental health, with implications for evidence-based urban planning and public health strategies [14,15,16].

Achieving high ecological validity in contemporary research requires capturing human responses during daily interactions in complex and dynamic urban environments [17]. This, however, presents methodological challenges. Multisensory and naturalistic paradigms, such as outdoor walks, must be adapted to ensure validity and comparability with laboratory-based protocols [18,19]. Furthermore, it remains technically demanding to synchronously measure environmental exposures (e.g., air quality, noise, microclimate, visual stimuli) alongside physiological responses (e.g., brain, cardiovascular, or autonomic signals) in real-world conditions.

### 1.1. Wearable Sensors and Outdoor Studies

Modelling and simulation approaches to urban comfort often underestimate the human dimension [20]. Human behaviour is strongly shaped by environmental context, and people in urban settings tend to act in ways that optimize comfort [21]. Furthermore, various features of the environment (e.g., the urban microclimate) that our comfort and wellbeing are dynamic—varying with the season [22] or time of day [23]. Hence, mobile and wearable sensors are becoming essential tools for assessing how microscale environmental conditions affect human behaviour [24], with a wide range of devices now available to monitor thermal, acoustic, or air quality parameters [25,26,27]. However, these environmental sensors are rarely integrated with physiological measures [27,28]. Conversely, many health-oriented studies still rely primarily on subjective assessments such as questionnaires, and only a minority incorporate portable biosensors [29]. Existing work has often focused on cardiovascular and respiratory outcomes of air quality exposure, while other environmental factors such as noise and temperature remain underexplored [28,29]. Some attempts have been made to integrate environmental and physiological sensing, such as combining meteorological monitoring with skin temperature recordings to assess thermal comfort [25], but these remain limited in scope.

Crucially, assessing mental states in urban contexts requires brain imaging methods, with electroencephalography (EEG) and, to a lesser extent, functional near-infrared spectroscopy (fNIRS) emerging as the most suitable techniques for outdoor use [11,13]. The EEG is generally favoured due to its millisecond temporal resolution and portability, which make it well suited for mobile applications; moreover, it is a well-established and extensively validated neuroimaging techniques [30,31]. The emergence of consumer-grade EEG devices has further expanded its use beyond laboratory settings, particularly in brain–computer interface research [19,32]. This availability has stimulated studies examining the performance of mobile EEG in moving participants, including analyses of signal quality and artifact management [33,34,35]. Some work has attempted to link mobile EEG recordings to environmental exposures—for example, treadmill-based paradigms [36] or outdoor walks with wireless EEG systems [37,38]. However, many of these studies rely on low-density EEG systems or setups vulnerable to motion artifacts, limiting their robustness in naturalistic conditions.

Growing interest in ecological validity has driven progress in mobile cognitive neuroscience, in parallel with advances in wearable recording technologies [16,18]. Yet significant challenges remain as urban environments are inherently complex and dynamic—requiring simultaneous and synchronized measurement of multiple influencing variables. Current studies rarely achieve this level of integration, which constrains their capacity to fully characterize human–environment interactions.

To address this gap, we developed the eMOTIONAL Cities Walker—a wearable, reconfigurable backpack system capable of synchronous acquisition of both physiological responses and environmental exposures during real-world urban exposure.

### 1.2. Scope and Objectives

This study was conducted within the framework of the eMOTIONAL Cities project (https://emotionalcities-h2020.eu/), a European Union Horizon 2020 Research and Innovation Action that investigates how natural and built urban environments shape human cognition and emotion through their neurobiological underpinnings. The project integrates advanced neuroscience methodologies, including neuroimaging and physiological monitoring with environmental sensing to generate robust evidence on the links between urban characteristics and individual well-being.

In this paper, we present the methodological foundations for conducting neuroscience research with freely moving participants in real-world urban environments. Specifically, we introduce a new multimodal wearable experimental platform that combines multiple environmental and biosensors, including electroencephalography (EEG) for assessment of mental state assessment, enabling both indoor and outdoor studies in naturalistic conditions. We also describe the implementation of a flexible experimental framework designed to ensure comparability, reliability, and reproducibility of data across laboratory and field paradigms.

By bridging laboratory-based and field-based approaches, this work contributes to the emerging domains of Environmental Neuroscience and Neurourbanism, by providing validated tools to capture the complexity of human experiences in urban spaces. Ultimately, the proposed framework supports the development of evidence-based strategies for urban planning and design aimed at promoting mental health and well-being.

**Figure 1:**
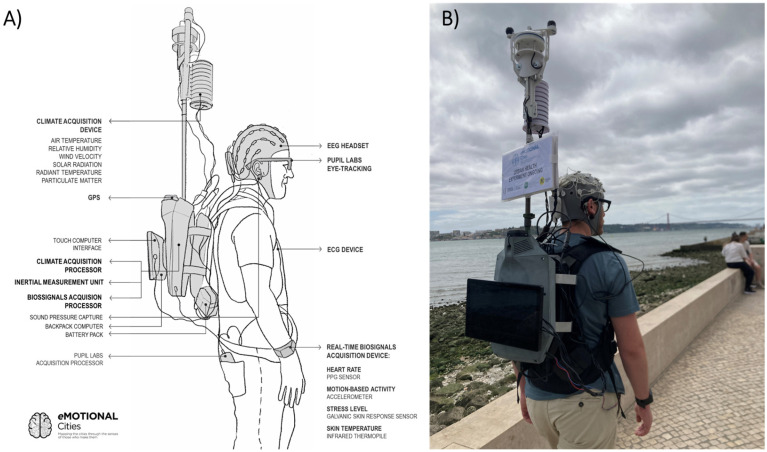
(**A**) Schematic of the eMOTIONAL Cities Walker backpack, illustrating the integrated devices and sensors of the data collection unit. (**B**) Side-view photograph of a participant wearing the backpack during an outdoor walk experiment.

The wearable data collection unit, termed the *eMOTIONAL Cities Walker*, is an ergonomically designed backpack apparatus developed to acquire multimodal data streams of data from both the user and the surrounding environment (Figure 1). At its core, the system employs a mobility-focused computer, the HP^®^ VR Backpack PC (HP Inc., Palo Alto, CA, USA), optimized for wearable use. The backpack’s design allows participants to walk naturally in outdoor environments while carrying the system comfortably. Dimension-wise, the backpack weighed approximately 9 kg when fully equipped, featured an adjustable height ranging from 86 cm to 118 cm, a depth between 23 cm and 27 cm, and a width of 28 cm. Dual hot-swappable external batteries ensure uninterrupted field operation for up to two hours. Its compact design, wireless connectivity, and portability make the system suitable for unobtrusive outdoor use with multiple biosensors and environmental sensors. To support continuous experimental oversight, a touchscreen interface was integrated, enabling real-time monitoring and interaction between the researcher and the system.

The eMOTIONAL Cities Walker comprises two main components—human-centred sensors and environmental sensors, supported by integration modules. These sensor systems were selected to work in a complementary manner, enabling the collection of data on human–environment interactions across multiple levels.

**Human-centered sensors:** the EEG was included to measure neural activity underlying cognitive functions (e.g., attention) and context-dependent mental states, including signals often associated with stress and restoration [17,31]. Eye-tracking provides geotagged, egocentric video data, enabling identification of environmental features attracting visual attention [39,40]. Combined with computer vision algorithms, eye-tracking facilitates object and landmark recognition in real-world settings [41]. Peripheral cardiovascular and autonomic sensors included electrocardiography (ECG), blood volume pulse (BVP), and galvanic skin response (GSR), providing indices of arousal and stress levels also used in other studies [42,43,44,45]. In addition, microphones were also considered by us to record ambient noise, a well-established environmental stressor

**Environmental sensors:** enable the estimation of thermal comfort metrics, such as the universal thermal climate index (UTCI) [46]. This index accounts for variables including air temperature, mean radiant temperature (via black globe temperature), relative humidity, and wind speed:

(1) $$\mathrm{UTCI}=f\left(T_{a},T_{g},RH,v\right)$$

$\mathrm{where}\;T_{a}=\mathrm{air}\;\mathrm{temperature},\;T_{g}=\mathrm{globe}\;\mathrm{temperature},\;RH=\mathrm{relative}\;\mathrm{humidity},\;\mathrm{and}\;v=\mathrm{wind}\;\mathrm{speed}.$ Additionally, real-time monitoring of particulate matter (PM) concentrations permits assessment of air quality and pollution exposure [47].

The various types of sensors were selected based on prior validation in urban and environmental neuroscience studies for myriad research questions and their utility in environmental neuroscience proven to be useful (Table 1); with portability, robustness, and compatibility being also prioritized for integration into a mobile platform. High sampling rates across modalities ensure the capture of both transient and sustained responses, facilitating advanced multimodal analysis of human experience in urban contexts.

## 2. System Concept and Design

### 2.1. Sensors for Human Physiology and Behavioural Signals

#### 2.1.1. Electroencephalogram (EEG)

EEG signals were acquired using a 32-channel Enobio^®^ system (Neuroelectrics^®^, Barcelona, Spain). The setup involved fitting a neoprene cap with dry electrodes positioned according to the international 10–20 system. Electrode cable sets were connected to a lightweight amplifier containing a rechargeable internal battery. The amplifier was secured with a hook-and-loop fastener and linked to the backpack computer via USB. Although the backpack can accommodate different EEG systems, outdoor use is constrained to portable, low-weight amplifiers. Dry electrodes were selected to enable rapid setup, despite their lower signal quality compared to wet electrodes [19,32]; however, the system also supports gel-based electrodes if required. EEG signals were streamed in real time to the Neuroelectrics^®^ Instrument Controller (NIC2) software (v2.0.11). Event markers were transmitted through the Lab Streaming Layer (LSL) to synchronize EEG with other sensor modalities [51].

#### 2.1.2. Peripheral Physiological Signals

Peripheral physiology was monitored using the E4 wristband (Empatica Inc., Boston, MA, USA). The device, worn on the participant’s non-dominant wrist to reduce motion artifacts, contains an internal rechargeable battery. The wristband integrates a photoplethysmography (PPG) sensor providing blood volume pulse (BVP), inter-beat interval (IBI), and heart rate (HR), alongside a three-axis accelerometer, skin temperature sensor, and electrodermal activity sensor. Data were transmitted wirelessly via Bluetooth to the E4 Streaming Server application and subsequently integrated into the Bonsai dataflow framework.

#### 2.1.3. Electrocardiogram (ECG)

The ECG recordings were obtained using the SparkFun^®^ AD8232 board (SparkFun Electronics, Niwot, CO, USA). Disposable gel electrodes (Ambu^®^ Blue Sensor^®^) were positioned on the torso following a three-lead configuration: right abdomen (red), right chest (white), and left chest (black). The electrodes were connected to the board with a 3.5 mm jack cable. This sensor provided more reliable cardiac signals than the wrist-worn PPG device, which often yielded missing data in the presence of arm movement and a looser watch placement. The AD8232 board was placed in the backpack’s electronics compartment and connected via USB to the processing unit.

#### 2.1.4. Eye-Tracker

Eye movements were captured with Pupil Invisible^®^ glasses (Pupil Labs GmbH, Berlin, Germany). After fitting, calibration was performed using a dedicated smartphone running the Pupil Invisible app, enabling rapid deployment. The glasses contain two inward-facing eye cameras and an outward-facing scene camera that recorded the participant’s visual field. The smartphone, carried in the participant’s pocket or strapped to the backpack, connected to the local Wi-Fi network to wirelessly transmit data to the backpack computer. Recorded variables included gaze position, blink events, fixation metrics, and synchronized video from the scene camera.

#### 2.1.5. Nine-Axis Inertial Measurement Unit (IMU)

Movement and orientation were measured using the BNO055 intelligent nine-axis absolute orientation sensor (Bosch Sensortec GmbH). The module integrates a 14-bit triaxial accelerometer, a 16-bit triaxial gyroscope, a triaxial geomagnetic sensor, and a 32-bit ARM Cortex-M0+ microcontroller running onboard sensor fusion algorithms. This device captured accelerations, heading changes, and stationary periods with higher temporal precision than Global Navigation Satellite System (GNSS) data. The sensor was directly interfaced with the backpack computer.

#### 2.1.6. Microphone

Binaural audio was recorded using OKM II Solo microphones (Soundman^®^, Berlin, Germany). Each microphone earpiece was inserted through the openings of the EEG cap to ensure stability and participant comfort.

### 2.2. Environmental Sensors

Environmental variables were recorded using sensors from Tinkerforge (Tinkerforge GmbH, Schloß Holte-Stukenbrock, Germany) and METER Group (Pullman, WA, USA). The Tinkerforge modules included the Humidity Bricklet (relative humidity), Thermocouple Bricklet (black globe temperature), Particulate Matter Bricklet (airborne particles), Sound Pressure Level Bricklet (acoustic intensity), Industrial Dual 0–20 mA Bricklet (irradiance), and Air Quality Bricklet (air pressure and air quality index). Complementary measurements of air temperature, wind velocity, and wind direction were obtained with the ATMOS 22 ultrasonic anemometer (METER Group), thereby enabling the characterization of microclimatic conditions during outdoor experiments. All these environmental sensors comprise the backpack’s mobile weather station module, whose specifications and data quality control measurements were assessed and compared to other mobile weather stations in another study [52].

### 2.3. Spatiotemporal Sensor Synchronization

Temporal synchronization of multimodal data streams was managed by Pluma, a Hardware Research Platform (Harp) compatible device. Harp is a standard for self-synchronizing hardware widely adopted in neuroscience for asynchronous real-time data acquisition and experimental control [53,54,55]. Synchronization between non-Harp-compatible devices was performed via Harp-timestamped TTL trigger pulses delivered by Pluma to all devices, both at the start and periodically throughout the experiment, to minimize desynchronization and timing drift. The temporal jitter between periodic random interval pulses is used as a reference for subsequent alignment of the data streams. All temporal alignment and synchronization code is available at https://github.com/emotional-cities/pluma-analysis (accessed on 17 November 2025). Spatial positioning was obtained via the ZED-F9R high-precision dead-reckoning GNSS module (u-blox, Thalwil, Switzerland). Each GNSS position sample was paired with a high precision Coordinated Universal Time (UTC) timestamp, which provided a common temporal reference. This timestamp was subsequently used to align and contextualize physiological, behavioral, and environmental data, ensuring accurate spatiotemporal integration across all sensor modalities (Table 2). Synchronization quality was assessed for all collected datasets as part of the quality control procedure described and illustrated in Figure S1.

### 2.4. Integration Software

**Figure 2:**
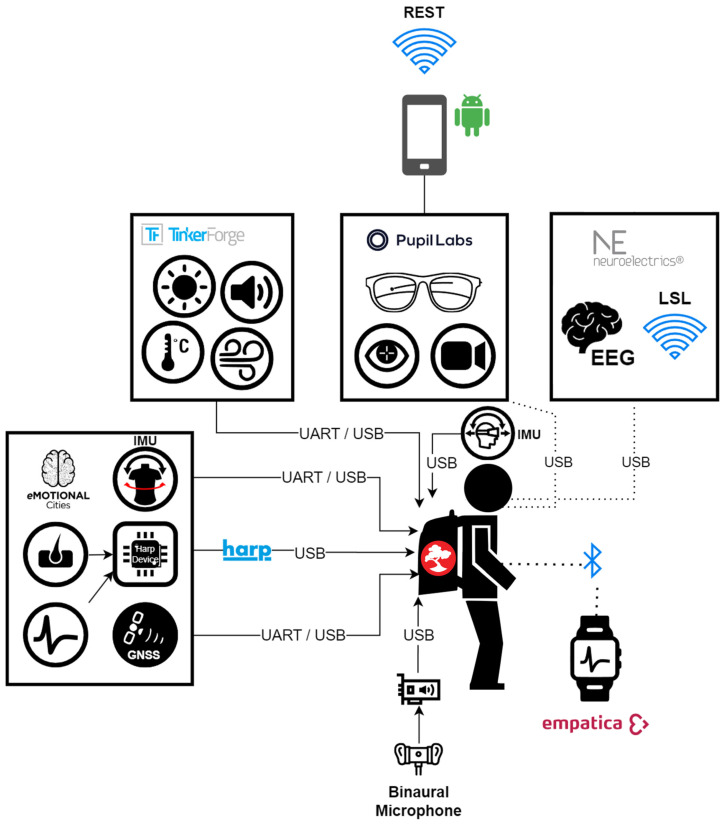
Schematic representation of the integration framework and communication protocols for each data stream. The Pupil Labs eye-tracking system required an external smartphone connected to the glasses via USB, which stored data locally and simultaneously transmitted it to the backpack computer over Wi-Fi. Empatica E4 data were streamed via Bluetooth. EEG signals were transferred to the backpack through either USB or Wi-Fi, with event synchronization managed using the LSL protocol. All other sensors communicated directly with the processing board through UART or USB interfaces, with temporal alignment provided by the Pluma Harp device.

To ensure spatiotemporal synchronization of multimodal data, all acquisition processes were integrated within a unified framework based on Bonsai, an open-source visual programming language for reactive data streams [56]. Standard interfaces were used to access each sensor, allowing seamless data routing and management. An overview of the integrated sensors and their communication protocols with the Bonsai software (v2.8.1) is presented in Figure 2. All experimental acquisition and control code, including descriptions and technical details of all system streams are available at https://github.com/emotional-cities/walker-experiments (accessed on 17 November 2025).

Bonsai generated random timing events, which were converted by the eMOTIONAL Cities Walker’s motherboard into Harp timestamps. These timestamps were routed as transistor–transistor logic (TTL) pulses to synchronize sensors capable of hardware-level alignment, including Tinkerforge modules, the Enobio^®^ EEG system, and the u-blox GNSS. Sampling of the nine-axis IMU was also hardware-triggered and Harp-timestamped by the motherboard.

This dual approach—hardware TTL pulses for compatible devices and software timestamping for others—ensured reliable temporal alignment across all data streams. Random timing events coupled with Harp timestamps enabled accurate reconstruction of temporal order, even in cases of missing data or dropped events. Ultimately, all sensor signals were synchronized to GNSS-derived UTC timestamps, providing a high-precision spatiotemporal reference for multimodal data integration.

## 3. Experimental Setting for Testing and Results

As a proof of principle, this section describes an experimental task in which the eMOTIONAL Cities Walker was deployed to collect multimodal data along predefined urban paths in Lisbon, Portugal.

### 3.1. Participants

Participants were recruited through convenience sampling using informal outreach strategies, including personal and professional networks (e.g., colleagues, friends, and acquaintances). Although this approach may have introduced some degree of selection bias, it was considered appropriate for this study’s aims and is not expected to have substantially influenced the present description. Eligibility criteria required participants to be ≥18 years of age, residing in Lisbon, fluent in Portuguese or English, and with no history of major psychiatric or neurological disorders. Volunteers were invited to participate in both indoor and outdoor experiments, although only three completed both. Each participant could repeat the task along different routes, with a maximum of three paths per individual. In total, 43 participants contributed to 60 walks (10 acquisitions per path). Participants received a voucher as compensation for their time. All subjects provided written informed consent, and the study protocol was approved by the Ethics Committee of the Institute of Geography and Spatial Planning, University of Lisbon (IGOT) (reference: ETHIC-07/2022).

### 3.2. Experimental Procedures and Stimuli

Participants were randomly assigned to one of six predefined paths, each corresponding to distinct urban environments in Lisbon. These paths were selected in a stakeholder workshop that brought together professionals from multiple domains to identify city areas capturing a wide range of urban characteristics [57].

Prior to the walk, participants were fitted with the multimodal backpack apparatus (see Section 2). Instructions emphasized that participants should behave as naturally as possible, abstracting from the experimental context to simulate an everyday walk. Each path was approximately 1 km in length. A researcher followed the participant at a discrete distance, providing verbal guidance if necessary.

**Figure 3:**
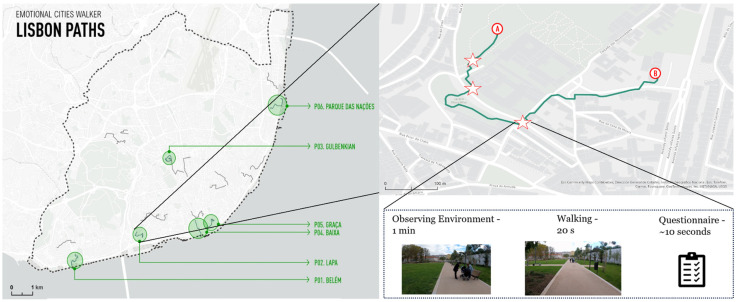
The outdoor walking task was conducted across six different paths in the city of Lisbon, which were chosen based on certain geographical and sociodemographic characteristics. Between the start (A) and end (B), each path also had some “checkpoints” (marked with stars)—where participants were asked to pause (observing the surroundings still for one minute), walk for 20 s and then pause again to answer several questions about the walking experience. The data gathered at these checkpoints was then compared to data acquired in a related laboratory-based experiment that used first-person video walks of the same section of the path.

Along each route, a few checkpoints were defined. These locations were also filmed for subsequent use in a complementary laboratory-based EEG experiment, designed to validate data across paradigms (full analysis reported separately). At each checkpoint, participants completed three tasks sequentially: (i) observing the surrounding environment while standing still; (ii) walking continuously for 20 s; and (iii) answering short questions to assess perceived naturalness, crowdedness, and subjective feelings of valence and arousal (Figure 3).

### 3.3. Sensor Reliability

The eMOTIONAL Cities Walker backpack proved overall reliable for outdoor multimodal data acquisition. Setup time was approximately 10 min per participant, which is efficient given the number and complexity of sensors. Participants across age groups completed the approximate 25 min walking protocol with minimal difficulty, highlighting the ergonomic suitability of the system.

**Figure 4:**
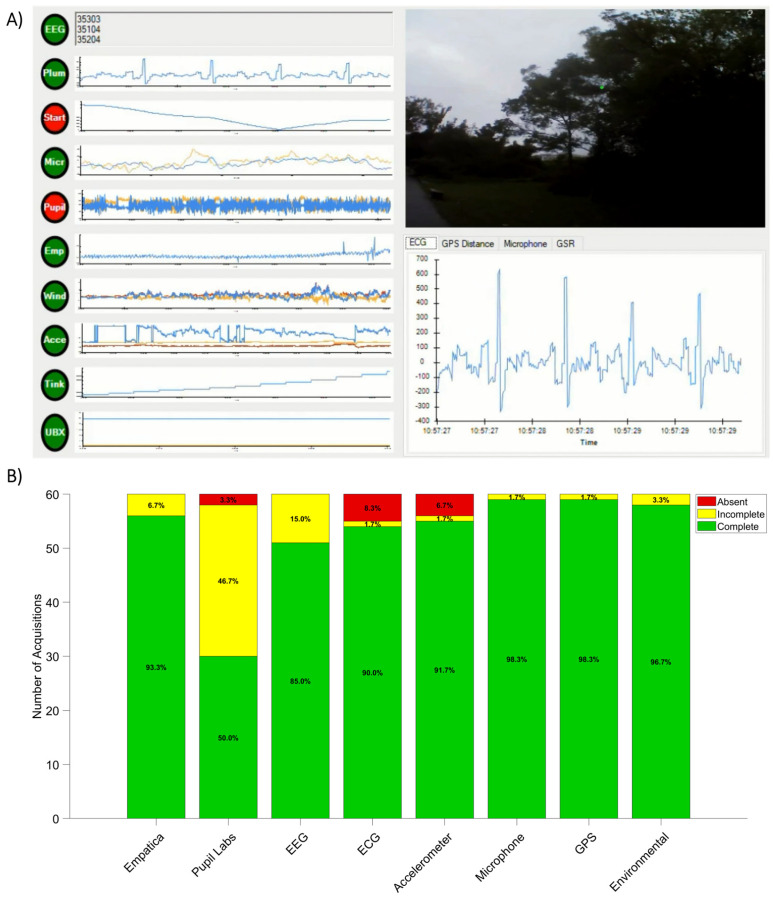
(**A**) Dashboard interface for real-time visualisation of multimodal data streams. The example illustrates a moment when the recording of Pupil Labs^®^ eye-tracking data (“Pupil”) failed, indicated by the corresponding red circle. Similarly, the red “Start” circle denotes that the participant has moved away from the initial GPS-calibration location. Green circles indicate that remaining sensors have adequate ongoing data acquisition. (**B**) Summary of sensor recording status across sixty data acquisition sessions conducted in Lisbon. For each path, individual sensors were classified as: (i) complete—data recorded throughout the entire path; (ii) incomplete—data recorded for only part of the path; or (iii) absent—no data recorded for that session.

Nevertheless, sensor data acquisition varied throughout the 60 sessions (Figure 4B). While most sensors functioned consistently, failures occasionally occurred due to: (i) mechanical wear of connectors with repeated use; (ii) cable strain and spurious disconnections during movement; and signal interference in outdoor settings. In practice, several hardware-related issues were encountered during data collection. The Empatica^®^ E4 wristband occasionally suffered from Bluetooth connectivity interruptions. The Pupil Labs^®^ Invisible eye-tracking glasses showed susceptibility to USB-C connectivity failures after extensive use, particularly at the connection port embedded in the glasses, and occasional data loss due to temporary Wi-Fi disconnections. The Enobio^®^ EEG system experienced similar mechanical wear at the connection interface between the amplifier and the backpack unit. Missing ECG data primarily resulted from suboptimal electrode placement—leading to detachment during walking; or, less frequently, from incorrect port connections. The causes of failure in the remaining sensors were more difficult to identify, as these devices operate within sealed internal modules whose connectivity is not directly accessible or handled by the researcher on site.

Whenever a sensor lost one or more samples during a given path, its corresponding data stream was labelled as incomplete and as complete otherwise. If a sensor failed to record any data throughout the walk (e.g., due to hardware malfunction, communication failure, or data corruption—see also example Figure 4A), it was labelled as absent (Figure 4B). Percent mean uptime was calculated for each sensor across all recording sessions to provide a fine-grained quantitative assessment of effective data acquisition. The environmental sensors within the Tinkerforge module achieved the highest reliability, with mean uptimes exceeding 99%. These were followed by the Empatica^®^ E4 wristband’s skin temperature and EDA streams, accelerometer data, and ECG signals, all showing mean uptimes of approximately 95%. The u-blox GPS module (elevation, longitude, and latitude) and the audio recordings each presented mean uptimes of around 84%—the reduction in the former primarily attributable to signal loss in underground areas or in locations surrounded by tall buildings. The Neuroelectrics^®^ Enobio EEG system exhibited a lower mean uptime of 74%, while the Empatica^®^ E4 heart rate stream showed the lowest value, with a mean uptime of about 16%.

### 3.4. Output Data Structure

For each acquisition, raw sensor outputs were stored in a dedicated folder. Electroencephalography (EEG) recordings were saved separately by the NIC software and subsequently integrated into the session dataset. All data streams were then consolidated into a spatiotemporal index using a Python-based (v3.11) pipeline (available at https://github.com/emotional-cities/notebooks, accessed on 17 November 2025), which enabled joint analysis of multimodal data.

**Figure 5:**
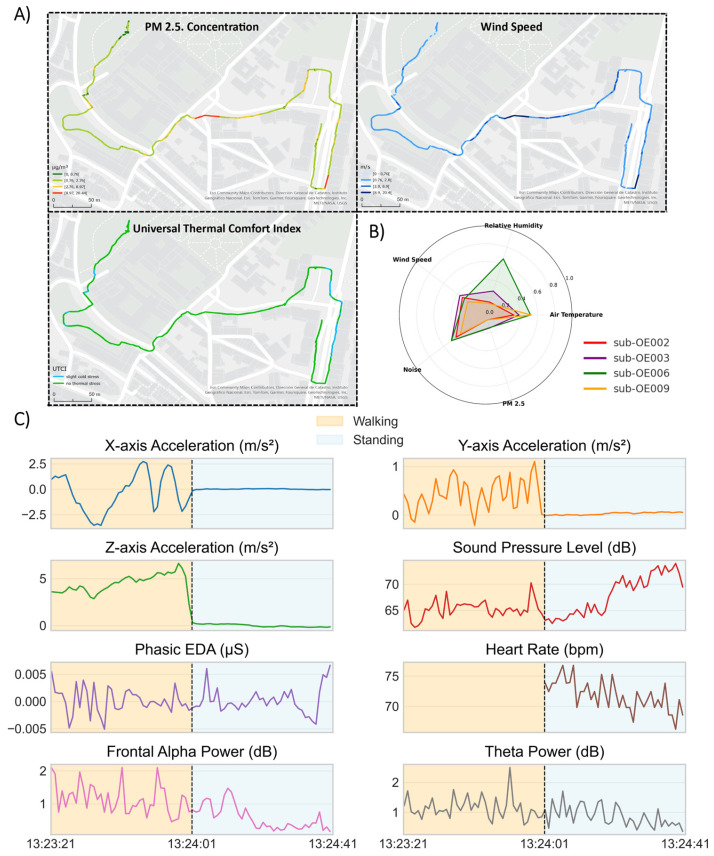
(**A**) Example of environmental data streams extracted from a single subject’s raw data for one and mapped to the corresponding path. Illustrated variables include PM2.5, UTCI, and wind speed, each plotted along the acquired GPS trajectory. (**B**) Radar chart displaying averages of normalized values (min-max) of climate sensor metrics from the same path across multiple participants, highlighting variability between sessions. (**C**) Derivative time series plots from one subject. The plots show different data streams, each re-sampled at 1 Hz, and synchronized from −40 to 40 s relative to the onset of the “stop walking” event. Phasic EDA values correspond to high-pass raw EDA values and EEG band power was computed with Welch’s method on two second windows with 50% overlap. The heart rate plot showcases data from the E4 wristband. The accelerometer and sound pressure levels streams were plotted directly as raw sensor outputs.

This framework allows environmental, physiological, and behavioural signals to be plotted alongside Global Positioning System (GPS) coordinates (Figure 5). Since GPS trajectories were consistent across sessions for each predefined path, the spatiotemporal structure facilitates direct comparative analyses both between participants and across sessions.

## 4. Discussion

We have described a novel wearable data collection unit that seamlessly integrates environmental and physiological sensors within a unified, synchronized, and portable system. Its innovation lies not only in the combined acquisition of biosensing and environmental data—enabling real-world investigations at the interface between urban environments, brain function, and human well-being, but also in the precise temporal synchronization achieved through a central computational unit implementing HARP-based alignment—ensuring millisecond-level correspondence across heterogeneous data streams.

One of the main advantages of equipping a powerful computer with a wearable backpack is the ability to acquire multimodal data through wired connections, which reduces the risk of data loss often associated with wireless communication (e.g., via Bluetooth). The apparatus is also highly modular and built around open-source hardware and software, granting researchers a level of flexibility rarely available in outdoor EEG studies within the field of Neurourbanism [58]. A particularly valuable feature is the use of Harp devices, which enable synchronization across sensors operating at different sampling rates—a longstanding challenge in multimodal integration [59]. With two fully charged batteries, the system supports approximately two hours of continuous recording, assuming all individual devices with internal batteries begin fully charged. In addition, the backpack’s computer itself possessed an internal battery, which extended acquisition time. In fact, the battery autonomy of individual sensors, such as the EEG amplifier, were usually the limiting factor for acquisition time. As such, the choice of external hardware needs to be considered when assessing the system’s overall autonomy. Furthermore, the backpack can be adapted for virtual reality tasks: the climate station may be removed to increase freedom of movement, while the system’s broad port selection allows compatibility with external devices such as VR headsets or GPU-accelerated computers for demanding VR modelling tasks.

The use of EEG is particularly valuable as it provides direct access to neural activity with millisecond temporal precision, offering insights into brain processes that underpin perception, cognition, and emotion. This makes EEG uniquely positioned to bridge neuroscience and urban environmental research, while also informing our understanding of mental health in real-world contexts. According to recent recommendations for the use of mobile EEG in outdoor settings [58], the Enobio^®^ 32-channel system offers several advantages: a lightweight amplifier, a cap with standard electrode positioning, and seamless integration with the LSL protocol. However, it also presents notable limitations: a lower channel count compared with the 64 channels often recommended for high-quality mobile EEG; occasional cable sway during walking; and mastoid reference electrodes that were prone to displacement. For this task, dry electrodes were preferred over gel electrodes to speed up preparation time and increase convenience for the participant, and while it was known that data quality would be greatly reduced, it proved to be an opportunity to understand the applicability of dry electrodes in the context of outdoor walks. Nonetheless, the 32-channel Enobio^®^ system also supports gel electrodes. Importantly, the modularity of the eMOTIONAL Cities Walker makes it sensor-agnostic, meaning it allows straightforward integration of alternative EEG systems and does not constrain the user to any particular EEG system (see [60,61] for a review on other devices).

Beyond EEG, the platform is designed to incorporate a comprehensive suite of multimodal body sensors, enabling a richer characterization of human–environment interactions. These include eye-tracking devices, which provide precise measures of visual attention; physiological sensors that capture cardiovascular metrics, electrodermal activity, and ECG signals to assess autonomic arousal and stress responses; and movement-tracking sensors that quantify accelerations, heading changes, and stationary periods with high temporal precision. Together, these complementary data streams enrich EEG-derived neural dynamics and support efforts to understand how cognitive, emotional, and behavioural states unfold in real-world environments [17,18]. This multimodal approach substantially strengthens ecological validity and opens new pathways for nuanced interpretations of how urban settings impact both mental and physiological well-being. It should be noted, however, that a thorough understanding of the interaction between environmental features and EEG data is not specifically addressed in this paper (as it will be the focus of future works within our group).

Although the outdoor paradigm aims to simulate a natural walk, participants are inevitably affected by various sources of discomfort, including the backpack’s weight, the sensation of EEG electrodes, and the social exposure in public spaces where photos may be taken. Nonetheless, a dropout rate of 0% was achieved across all 60 sessions. In the qualitative analysis of post-walk interviews, 16 participants mentioned the backpack in their responses, with 14 of these references being related to perceived discomfort associated with its volume or visibility—i.e., drawing attention in public spaces, rather than to any significant physical strain or pain. Moreover, most sensors are sensitive to movement, leading to degraded signal quality. The EEG signal is particularly highly susceptible to motion artefacts, with signal distortions that often exceed the amplitude of actual brain activity. Mitigation strategies include instructing participants to maintain steady gait, using active electrodes (gel-based or dry electrodes designed for better hair penetration), and employing higher-density EEG systems with more channels that benefit more from advanced preprocessing tools. Gait dynamics contribute significantly to periodic cable sway that can pull on the EEG electrodes and cause huge artifacts. Solutions to this problem include the use of strain-relief connectors and wireless alternatives. Likewise, ECG and EDA sensors are sensitive to movement artifacts. For example, wrist-based EDA sensors require a tight fit on the participant’s wrist and minimal arm sway to guarantee correct signal acquisition, which incidentally explains why the 3-lead ECG exhibited fewer data losses than the Empatica^®^ E4 heart-rate stream during field recordings.

It should be noted, however, that the primary aim of this study was not to directly evaluate signal quality in ambulatory conditions, but rather to demonstrate the successful integration of multiple sensing devices within a unified acquisition framework. Validation of signal quality and reliability for the individual sensors in relatively similar settings has been extensively reported elsewhere (see, for example [62] for the Empatica^®^ E4 wristband and [63] for the Neuroelectrics^®^ Enobio 32-channel EEG system with dry electrodes).

Despite these challenges, the eMOTIONAL Cities Walker Backpack demonstrates the feasibility of integrating complex, multimodal data streams essential for investigating brain–environment interactions in naturalistic settings. A key strength of the system lies in its use of redundancy to increase reliability and safeguard against individual sensor failure. Among all data types, spatiotemporal streams are the most critical, as they provide the framework for synchronizing all other signals. GPS plays a central role in spatial referencing, though its accuracy can be compromised in dense urban environments such as tunnels or areas with tall buildings. In such cases, video footage from the eye-tracking device’s external camera offers a valuable fallback for inferring spatial context. Temporal synchrony—ensured by Harp devices—is irreplaceable, as it anchors all data streams to a precise timeline.

Looking ahead, this apparatus sets a new benchmark for mobile neuroscience platforms. Future developments should prioritize enhancing participant comfort and discretion to further increase ecological validity and participant compliance in outdoor research, while also expanding interoperability with emerging sensing technologies.

## Figures and Tables

**Table 1:** Some examples of previous applications and outcomes of different sensor modalities in environmental psychology or neurourbanism research.

Modality	Use Cases	Outcomes
EEG	Band power activity and time-domain neural responses associated with emotional states or cognitive tasks in urban environments [13,39].	Increases in global alpha and theta power have been associated with relaxation states, particularly in naturalistic environmental settings [13].
Eye-tracker	Eye-tracking metrics such as fixations, saccades, blink rate, and scanpath length provide valuable indices of attentional processes in real-world environments [40].	In spatial navigation tasks, increased visual attention to salient landmarks—as reflected in gaze fixations—enhances self-localization performance in urban environments [41].
Cardiovascular	Cardiovascular metrics such as heart rate or blood volume pulse are widely used as real time indicators of autonomic stress responses in urban contexts [42].	Wearable monitoring has revealed significant heart rate differences between wetland and urban settings, indicating measurable physiological benefits of exposure to natural environments [43].
Electrodermal activity (EDA)	The EDA provides a reliable index of emotional arousal, allowing researchers to capture how different environmental features elicit affective responses [44]	Wearable sensors for EDA have identified acute moments of stress during city walks, providing fine-grained insights into how urban environments impact human well-being [45]
Environmental	Identification of emotional “micro-spaces” within the built environment that contribute to better wellbeing [48,49]	Air pollution, high temperature and inadequate light intensity have been shown to modulate physiological responses—acting as potential urban stressors [48,49,50]

**Table 2:** Summary of the sensors in the eMOTIONAL Cities Walker.

	Name	Brand	Data Type	Units	Sampling Rate
**Biosensors**	Enobio^®^ 32 headset	Neuroelectrics^®^	EEG	μV	500 Hz
	Pupil Invisible^®^	Pupil Labs GmbH	World Camera	Image Frame	32 Hz
			Gaze	Pixels	250 Hz
	AD8232 Heart Rate Monitor	SparkFun^®^	ECG	mV	1000 Hz
	Binaural OKM II Solo microphones	Soundman^®^	Audio	dBV/Pa	44,100 Hz
	E4 wristband	Empatica Inc.	Heart rate	bpm	1.56 Hz
			Blood Volume Pulse	mmHg	64 Hz
			Skin Temperature	°C	4 Hz
			Electrodermal Activity	μS	
	BNO055 9-axis IMU	Bosch Sensortec GmbH	Orientation	degrees	50 Hz
			Gyroscope		
			Magnetometer		
			Accelerometer	m/s^2^	
			Gravity		
Spatiotemporal Sensors *	Harp Clock	Harp	Harp Timestamp	μs	31,250 Hz
	GNSS ZED-F9R	u-blox	Latitude	DDªMIN’SEC” DIRECTION	1 Hz
			Longitude		
			Altitude	meters	
			Time	hh:mm:ss	
**Environmental Sensors**	PTC Bricklet	Tinkerforge	Temperature	°C	100 Hz
	Industrial Dual 0–20 mA Bricklet		Irradiance	mA	
	Thermocouple Bricklet		Black Globe Temperature	°C	
	Particulate Matter Bricklet		Particulate Matter	μg/m^3^	
	Sound Pressure Level Bricklet		Sound Pressure Level	dB(A)	
	Humidity Bricklet		Humidity	%RH	1 Hz
	Air Quality Bricklet		Air Pressure	hPa	
	Atmos 22	METER Group	North Wind Speed	m/s	2 Hz
			East Wind Speed		
			Gust Wind		

* The synchronization between GPS spatial signal and the Harp clock signal is described in detail in Section 2.3.

## Data Availability

The raw data supporting the conclusions of this article will be made available by the authors on request.

## Supplementary Materials

The following supporting information can be downloaded at: https://www.mdpi.com/article/10.3390/s25237163/s1, Figure S1: Example quality control metrics collected to assess spatiotemporal synchronization for every acquisition session.
