# Supplementary material for: Advancing Mobile Neuroscience: A Novel Wearable Backpack for Multi-Sensor Research in Urban Environments
Source: Sensors (Basel). 2025 Nov 24;25(23):7163. doi: 10.3390/s25237163 (PMC12694519; doi:10.3390/s25237163)
Supplement: Supplementary file 1 [file sensors-25-07163-s001.zip › sensors-3930843-supplementary.pdf]

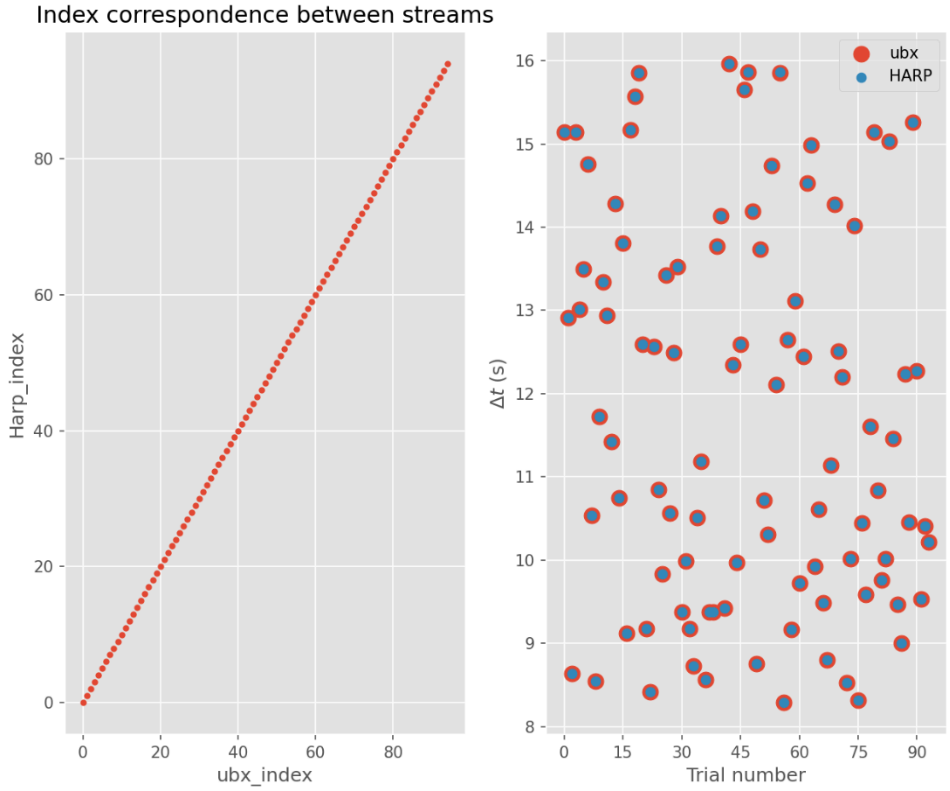

Supplemental Figure S1. Example quality control metrics collected to assess spatiotemporal synchronization for every acquisition session. A synchronization pulse is periodically sent at pseudo-random intervals from the Harp clock board to the ZED-F9P GNSS module. Each pulse is automatically timestamped with the current GNSS time and sent to the host computer via the UBX protocol. **Left)** index correspondence between UBX TIM-TM2 logging sync pulses received by the GNSS module and Harp messages reporting sync pulses sent by the clock board. Perfect diagonal means all emitted sync pulses were received and timestamped by the GNSS module. **Right)** summary statistics showing sync pulse alignment. Y-axis shows the time interval between consecutive pulses. Uniform tiling of intervals confirms pseudo-random distribution. The delta between TIM-TM2 timestamps and Harp timestamps for each pulse is shown to match exactly. Using these correspondences we build a linear regression model (r^2 = 0.99) used to map all GNSS timestamps to Harp time. A crucial aspect of our system is precise spatiotemporal synchronization of all data. To do this we synchronize all timestamps to Harp clock and tag all measurements with periodic GNSS positioning updates. In Supplemental Figure 1 we show an example of how we validate the synchronization between the GNSS module and the Harp clock for a single acquisition session. Corresponding quality control and benchmarks were performed between every pair of independent clocks integrated in the system.
